# Contrasting Changes Caused by Drought and Submergence Stresses in Bermudagrass (*Cynodon dactylon*)

**DOI:** 10.3389/fpls.2015.00951

**Published:** 2015-11-10

**Authors:** Tiantian Ye, Haitao Shi, Yanping Wang, Zhulong Chan

**Affiliations:** ^1^Key Laboratory of Plant Germplasm Enhancement and Specialty Agriculture, Wuhan Botanical Garden, Chinese Academy of SciencesWuhan, China; ^2^University of Chinese Academy of SciencesBeijing, China

**Keywords:** bermudagrass, drought stress tolerance, sumbergence stress, Proteomic analysis, reactive oxygen species, carbohydrate metabolism

## Abstract

In this study, we investigated the mechanisms by which bermudagrass withstands the drought and submergence stresses through physiological, proteomic and metabolomic approaches. The results showed that significant physiological changes were observed after drought treatment, while only slight changes after submergence treatment, including compatible solute contents, ROS levels and antioxidant enzyme activities. Proteomics results showed that 81 proteins regulated by drought or submergence treatment were identified by MALDI-TOF-MS. Among them, 76 proteins were modulated by drought stress with 46 increased abundance and 30 decreased abundance. Forty-five showed abundance changes after submergence treatment with 10 increased and 35 decreased. Pathway enrichment analysis revealed that pathways of amino acid metabolism and mitochondrial electron transport/ATP synthesis were only enriched by drought treatment, while other pathways including photosynthesis, biodegradation of xenobiotics, oxidative pentose phosphate, glycolysis and redox were commonly over-represented after both drought and submergence treatments. Metabolomic analysis indicated that most of the metabolites were up-regulated by drought stress, while 34 of 40 metabolites contents exhibited down-regulation or no significant changes when exposed to submergence stress, including sugars and sugar alcohols. These data indicated that drought stress extensively promoted photosynthesis and redox metabolisms while submergence stress caused declined metabolisms and dormancy in *Cynodon dactylon*. Taken together, the quiescence strategy with retarded growth might allow bermudagrass to be adaptive to long-term submerged environment, while activation of photosynthesis and redox, and accumulation of compatible solutes and molecular chaperones increased bermudagrass tolerance to drought stress.

## Introduction

Drought and flooding are major abiotic factors limiting plant growth and development which happened from time to time worldwide. Under drought stress condition, limited water supply greatly decreases leaf water content and causes tissue dehydration which is characterized by extensive changes at physiological, biochemical, molecular, and cellular levels (Ashraf, 2010; Fleury et al., 2010). Drought tolerance is a very complex trait depending on severity of the drought, plant developmental stage as well as the stress duration (Zhu, 2002). Drought stress induces the accumulation of the plant hormone abscisic acid (ABA), which leads to stomatal closure for maintaining water status in plant cells under water-deficit conditions (Ren et al., 2010; Zhao et al., 2013).

Flooding is another form of water stress that results from excess water, which affects about 10% of the global land area. Flooding, including waterlogging and submergence, can negatively affect plant growth and crop production (Setter and Waters, 2003). Waterlogging is defined as the saturation of the soil with water around the roots, while submergence describes the condition in which the whole plant is completely covered by water (Liu and Jiang, 2015). Under submergence environment, gases such as O_2_, CO_2_, and ethylene diffuse very slowly in water and the cellular O_2_ level decreases and inhibits aerobic respiration (Gibbs and Greenway, 2003; Fukao and Bailey-Serres, 2004). Despite knowledge of adaptive mechanisms to drought, understanding of the mechanisms behind plant response to submergence is very limited. Plants develop different strategies in response to submergence. Recent studies showed that many genes were involved in submergence responses (Gonzali et al., 2005; Xu et al., 2006; Hattori et al., 2009). In rice, flood-tolerant cultivars invoke a quiescence strategy that is controlled by transcription factors *SUB1*. *SUB1A* is induced by ethylene under submergence condition and negatively regulates expression of *SUB1C*, leading to repressed carbohydrate metabolism and retarded cell elongation. Flood-susceptible rice cultivars avoid submergence via activation of *SUB1C* expression which is promoted by gibberellic acid (GA) and is associated with rapid degradation of carbohydrate reserves and enhanced elongation of leaves and internodes (Bailey-Serres and Voesenek, 2008; Xu et al., 2006).

Grass plants were exposed to either drought or flooding conditions frequently. Several groups reported growth changes of perennial grass under waterlogging condition. The results showed that waterlogging reduced shoot and root dry weight in cool-season grass species including creeping bentgrass (*Agrostis stolonifera*) (Huang et al., 1998; Jiang and Wang, 2006) and Kentucky bluegrass (*Poa pratensis*) (Wang and Jiang, 2007). However, waterlogging stimulated plant growth in the tolerant warm-season grass species such as knotgrass (*Paspalum paspaloides*) and spiny mudgrass (*Pseudoraphis spinescens*), while inhibited the growth in the intolerant seashore paspalum (*Paspalum vaginatum*) and centipedegrass (*Eremochloa ophiuroides*) (Zong et al., 2015). Comparative physiological analysis showed that submergence caused greater damage in perennial ryegrass (*Lolium perenne*) than waterlogging, increased greater reductions in leaf chlorophyll and total carotenoid concentrations (Liu and Jiang, 2015). The responses of diverse perennial ryegrass accessions to submergence and their recovery following de-submergence were also reported by the same group. The results indicated that large phenotypic variations in leaf color, plant height, and growth rate were observed under submergence condition (Yu et al., 2012).

As one of the most important warm-season turfgrasses, bermudagrass (*Cynodon dactylon*) exhibited high tolerance to several abiotic stresses including drought and submergence. Recently, we identified bermudagrass varieties that were differing in drought tolerance. Comparative physiological analysis showed that changes of water status, osmolyte accumulation and antioxidant defense system might be contributed to the natural variation of drought tolerance between bermudagrass varieties (Lu et al., 2009; Shi et al., 2012). Net CO_2_ assimilation and stomatal conductance to water vapor were inhibited by drought stress (Carmo-Silva et al., 2008a). However, activity of the enzymes involved in the assimilation of CO_2_ did not show significant change by drought treatment in three C4 grasses of different subtypes (Carmo-Silva et al., 2008b). Proteomic profiling identified 39 and 54 proteins that were regulated by drought stress in different bermudagrass cultivars, respectively (Zhao et al., 2011; Shi et al., 2014). Exogenous application of small molecules increased drought stress tolerance of *C. dactylon*. Totally 36 and 76 proteins were induced by polyamine and melatonin, respectively, in *C. dactylon* based on proteomics approach (Shi et al., 2013, 2015b). Additionally, the macroarray and RNA sequencing analyses identified stress-responsive candidate genes from *C. dactylon* (Kim et al., 2009; Shi et al., 2015a). Overexpression of a *C. dactylon* stress-responsive nuclear factor Y gene (*Cdt-NF-YC1*) in rice resulted in increased tolerance to drought and salt as well as increased sensitivity to ABA (Chen et al., 2015).

As indicated above, responses of *C. dactylon* to drought condition have been well characterized by several groups. However, limited information is available for the responses of *C. dactylon* to submergence condition. Field survey data in the water level fluctuation zone of the Three Gorges Reservoir in China demonstrated that most original vegetation disappeared due to winter flooding for up to 6 months, while perennials including *C. dactylon* could tolerant deep and long-term flooding condition (Ye et al., 2013; Wang et al., 2014). Physiological analysis showed that submergence increased antioxidant enzyme activities, but decreased total soluble carbohydrate and starch contents (Tan et al., 2010). However, the detailed proteomic and metabolomic changes in *C. dactylon* in response to sumbergence are largely unknown. Moreover, studies to directly compare contrasting responses after drought and submergence in *C. dactylon* were lacking and the underlying mechanisms remained elusive. Here comparative proteomics and metabolomics approaches were applied to investigate the mechanisms by which bermudagrass withstands the drought and submergence stresses. The results showed that drought stress extensively promoted photosynthesis and redox metabolisms while submergence stress caused declined metabolisms and dormancy in *C. dactylon*. Therefore, growth of *C. dactylon* was severely inhibited by drought, but completely by submergence, indicating different strategies resulted in contrasting growth adaption in *C. dactylon* in response to drought and submergence stresses.

## Materials and methods

### Plant materials and growth conditions

The bermudagrass seeds Yukon were kindly provided by American Seed Research of Oregon Company. After 3 days of stratification at 4°C in the dark, the seeds were sown in the flowerpot filled with soil in the greenhouse and were grown under long-day lighting conditions (16 h light/8 h dark), with about 65% relative humidity at 25 ± 2°C and light irradiance of about 150 μmol quanta m^−2^s^−1^ per day. The plants were irrigated with nutrient solution twice every week.

### Experimental design of stress treatments

To compare the differences of bermudagrass responses to drought and submergence, 21-day-old seedlings were subjected to control condition and stress conditions. For drought treatment, water was withheld for 21 d. For submergence treatment, plants were fully submerged in larger plastic containers (60 × 40 × 27 cm) for 21 d. The survival rate of stressed bermudagrass was recorded at 7 d after re-watering (for drought treatment) or de-submergence (for submergence treatment). The leaf samples were collected at 0, 7, 14, 21 days after control and stress treatments for physiological indexes analyses. The leaf samples at 14 days subjected to control and stress conditions were harvested for proteomic and metabolomic assays based on measured electrolyte leakage data (Figure S1). For each independent experiment, every plant sample was extracted from at least 30 bermudagrass plants. All the experiments in this study were repeated three times.

### Determination of leaf water content (LWC) and electrolyte leakage (EL)

For the relative LWC analysis, the leaf samples were harvested from at least 30 independent lines of different treatments at different time points (0, 7, 14, and 21 days). The fresh weight (FW) was weighed immediately after collection, and the dry weight (DW) was quantified after incubation for 16 h at 80°C, and the LWC (%) was measured as (FW-DW)/FW × 100 (Shi et al., 2012, 2014).

EL was determined from detached leaves, which were collected from at least 30 plants each treatment (about 0.2 g), The detached leaves were placed in 50 ml tubes containing 15 mL deionized water. After gently shake at room temperature for 6 h at 150 rpm, the initial conductivity was determined. The fully releasing conductivity was measured after boiling at 121°C for 20 min using previous samples. The conductivity was measured using a conductivity meter (Leici-DDS-307A, Shanghai, China). The percentage of electrolyte leakage was determined as the ratio of the initial conductivity to fully releasing conductivity as described previously (Shi et al., 2012, 2014).

### Quantification of sucrose and soluble total sugars

The sucrose and soluble total sugars were measured using the method as previously described by Shi et al. (2012). The sucrose content and soluble total sugar content of samples were measured at 480 nm of absorbance and calculated by using the standard curve with known concentration of sucrose and glucose.

### Measurement of malondialdehyde (MDA) and proline contents

The MDA content in control and stressed plant samples was extracted using thiobarbituric acid (TBA) regent and boiled at 100°C for 20 min as previously described by Yang et al. (2010). After cooling to room temperature and centrifugation at 15,000 g for 10 min, the supernatant was quantified at 450, 532, and 600 nm of absorbance with a spectrometer. The MDA concentration can be estimated through the following formula (μmol l^−1^) = 6.45(A_532_ - A_600_) – 0.56A_450_.

Proline content was measured by a spectrometric method using known concentration of L-proline to form standard curve. Briefly, 0.25 g leaf samples were grinded to power and then extracted in 3% (w/v) sulfosalicylic acid for 10 min at 100°C, then 2 ml ninhydrin reagent and 2 ml glacial acetic acid were added to the 2 ml extraction solution. The mixed solution was boiled at 100°C for 40 min. After cooling to room temperature, the proline level of sample was measured absorbance at 520 nm and calculated according to the standard curve as described previously (Shi et al., 2012).

### Determination of ROS accumulation and antioxidant enzyme activities

The protein concentration was quantified using the Bradford method (Bradford, 1976). For H_2_O_2_ content analysis, supernatant of the plant extracts and 0.1% (w/v) titanium sulfate regent [in 20% (v/v) H_2_SO_4_] were mixed at 1/1 (v/v) to precipitate the peroxide—titanium complex. The absorbance of solution was quantified at 410 nm. For the ${\text{O}}_{2}^{∙}$ - content assay, a plant ${\text{O}}_{2}^{∙}$ - ELISA Kit (Dingguo, Beijing, China) was used. The absorbance was quantified at 405 nm.

The catalase (CAT, EC chsdateIsROCDateFalseIsLunarDateFalseDay30 Month12Year18991.11.1.6), glutathione reductase (GR, EC 1.6.4.2) and peroxidase (POD, EC 1.11.1.7) activities were determined using CAT Assay Kit (Beyotime, Shanghai, China), GR Assay Kit (Beyotime, Shanghai, China) and Plant POD Assay Kit (Nanjing Jiancheng Bioengineering Institute, Nanjing, China), respectively, as described previously (Shi et al., 2012).

### Protein extraction and 2-DE

Total protein was extracted according to the previously described method with slight modifications (Chan et al., 2007). Briefly, 1 g frozen powder from plant leave were homogenized extensively with 5 ml of pre-cooled homogenization buffer [20 mM Tris-HCl (pH 7.5), 1.05 M sucrose, 10 mM EGTA, 1 mM DTT, 1 mM PMSF and 1% (v/v) Triton X-100] on ice, and centrifuged at 10,000 g for 30 min at 4°C. The supernatant was then mixed with equal volume of Tris-HCl (pH 7.8) buffered phenol. After centrifugation at 10,000 g for 30 min at 4°C, the above phenol phase was mixed with five volumes of ice-cold saturated ammonium acetate in methanol overnight at −20°C. The total proteins were collected through centrifugation was stored at −80°C or dissolved in the lysis buffer [7 M urea, 2 M mithiourea, 4% (w/v) of 3-[(3-cholamidopropyl)-dimethylammo-nio]-1-propane sulfonate (CHAPS), 65 mM DTT and 0.2% (w/v) of carrier ampholyte (pH3.5–10)]. After dissolving extensively and centrifugation, the protein supernatant was quantified through the Bradford's method (Bradford, 1976).

The 2-DE was performed as described by Shi et al. (2013) with minor modification. Briefly, 1 mg of total proteins was applied onto an immobilized pH gradient (IPG) strip (17 cm, pH 4–7, Bio-Rad, USA) and rehydrated extensively at room temperature overnight. The next day, the rehydrated strips were transferred to isoelectric focus (IEF) in the Protein IEF system (Bio-Rad, USA). The conditions of IEF and SDS-PAGE were the same as described by Shi et al. (2012).

### Gel image analysis and protein spot identification by MALDI-TOF-MS

The 2-D gels were stained in Coomassie brilliant blue R250 staining buffer for 4 h and distained overnight. After scanning with an EPSON PERFECTION V700 PHOTO scanner (Epson), the protein spot images of 2-D gel were analyzed using PDQuest 2-DE Analysis Software (BIO-RAD, USA). Protein spots with more than 2-fold abundance change were used for trypsin digestion and MALDI-TOF-MS analysis with AXIMA-CFR plus (Shimadzu Biotech, Kyoto, Japan) as reported by Shi et al. (2013). MASCOT software (Mascot Wizard chsdateIsROCDateFalseIsLunarDateFalseDay30Month12Year18991.2.0, Matrix Science Ltd., http://www.matrixscience.com) was used to analyze the MS data. Since bermudagrass is an un-sequenced species, the homologous proteins were blasted against sequenced plant species. In the searching process against NCBInr and Swiss-Port protein sequence databases, peptide masses were assumed to be monoisotopic, and 100 ppm was used as mass accuracy, and one missing cleavage site was the maximum, and modifications were also considered. The minimum score of 43 and the minimum sequence coverage of 6% in MOWSE analysis were used to keep the confidence of the identification results.

### Quantification of metabolites

The metabolites extraction and derivatization were performed as described by Lisec et al. (2006) and Sanchez-Villarreal et al. (2013). The metabolites were then determined using GC-TOF-MS (Agilent 7890A/5975C, CA, USA) according to the procedure of Lisec et al. (2006). For GC-TOF-MS, 1 mL of derivatizated extract was injected into a DB-5MS capillary (30 m × 0.25 mm × 0.25 mm, Agilent J&W GC Column, USA). The metabolites were identified based on retention time index specific masses, via comparing with reference spectra in mass spectral libraries (NIST 2005, Wiley 7.0). After metabolite identification, quantification of metabolites was performed based on the pre-added ribitol in the process of metabolite extraction that was used as an internal standard.

### Cluster analyses

Hierarchical cluster analysis was performed using CLUSTER program (http://bonsai.hgc.jp/~mdehoon/software/cluster/) (de Hoon et al., 2004). The resulting tree figures were displayed using the software package and Java Treeview (http://jtreeview.sourceforge.net/). The pathway graph of carbon metabolism was obtained from KEGG (http://www.genome.jp/kegg/pathway.html). The proteins with different abundance changes were classified using the Classification SuperViewer Tool (http://bar.utoronto.ca/ntools/cgi-bin/ntools_classification_superviewer.cgi) (Provart and Zhu, 2003) and functional categories of every protein were assigned using MapMan (http://mapman.mpimp-golm.mpg.de/general/ora/ora.html) (Thimm et al., 2004). Normalized frequency (NF) of each functional category was assayed as sample frequency of each category in this experiment/background frequency of each category in genome.

### Statistical analysis

All the experiments in this study were conducted three times, and the data shown are the means ± SEs, while the mean is the average of three replicates. For each independent experiment, every plant sample was extracted from at least 30 bermudagrass plants. Different letters above the columns in every figure indicate significant differences at *P* < 0.05 (according to Duncan's method).

## Results

### Drought severely while submergence completely inhibited growth of bermudagrass

**Figure 1:**
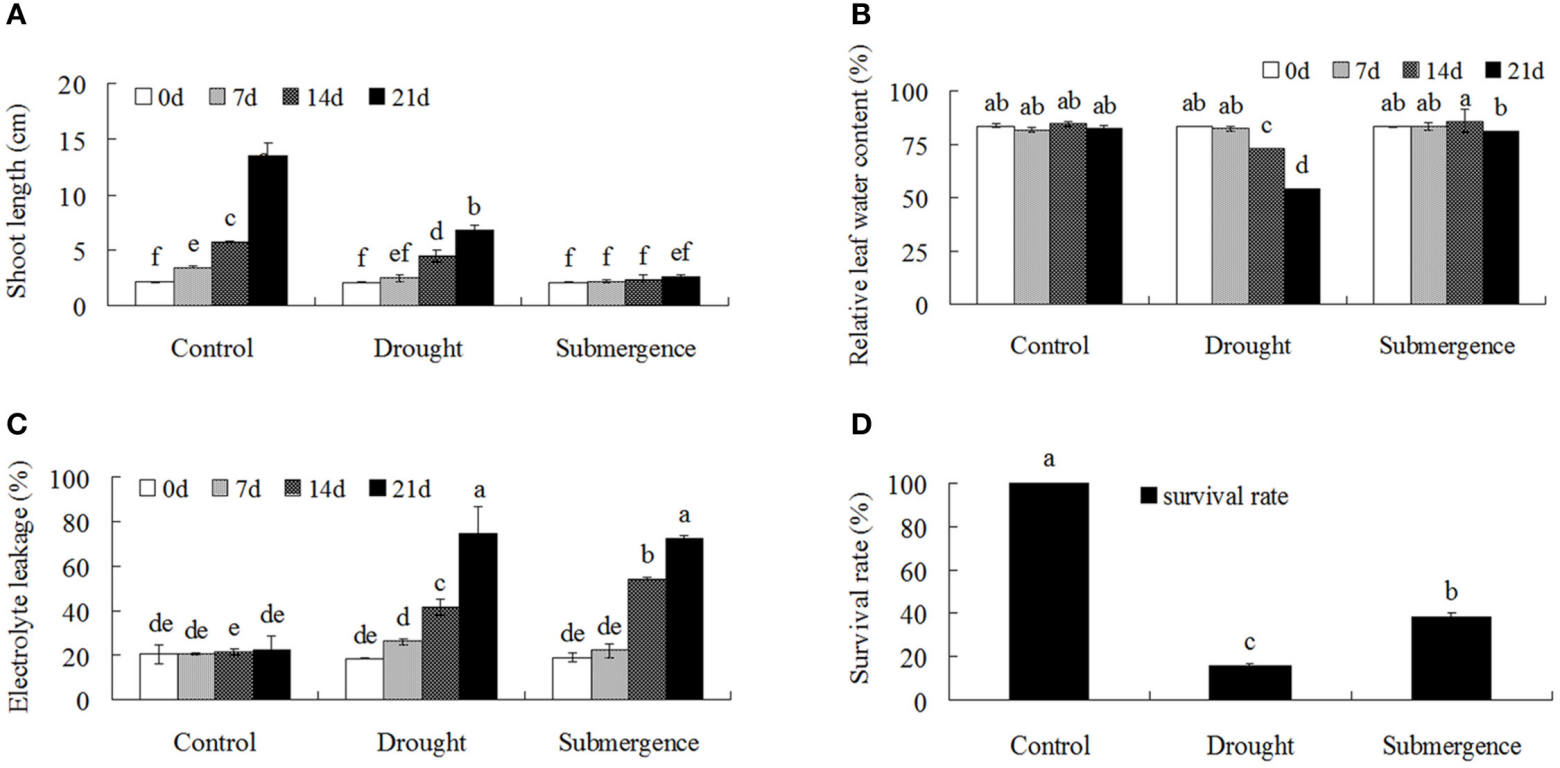
**Comparison of physiological responses to drought and submergence in bermudagrass**. Shoot length **(A)**, Relative LWC **(B)**, EL **(C)** of bermudagrass under control and stressed condition at designated time intervals. **(D)** Survival rate of bermudagrass after 21 days of control and stress treatments. The data represent the means of three independent experiment ± SE, and data followed by different letters are significantly different from each other at *P* < 0.05 according to Duncan's method.

Bermudagrass seedlings under control condition grew well with the shoot length from 2.1 cm at 0 d to 13.5 cm at 21 d after treatment. Drought severely and submergence completely inhibited seedling growth. The shoot length only reached 6.8 cm at 21 d under drought condition, while remained 2.6 cm after submergence treatment, which was only 19% of control seedlings (Figure 1A). Relative leaf water content decreased significantly after 14 and 21 d of drought treatment, but no differences were observed under submergence condition (Figure 1B). Both drought and submergence treatments significantly increased electrolyte leakage, resulting in increased cell membrane damages. At 21 after treatments, less than 16% seedlings survived under drought condition, while 38.6% seedlings survived under submergence condition. These results indicated that both drought and submergence treatments caused severe cell membrane damages and greatly inhibited bermudagrass growth.

### Contrasting effect of drought and submergence on compatible solute accumulation

**Figure 2:**
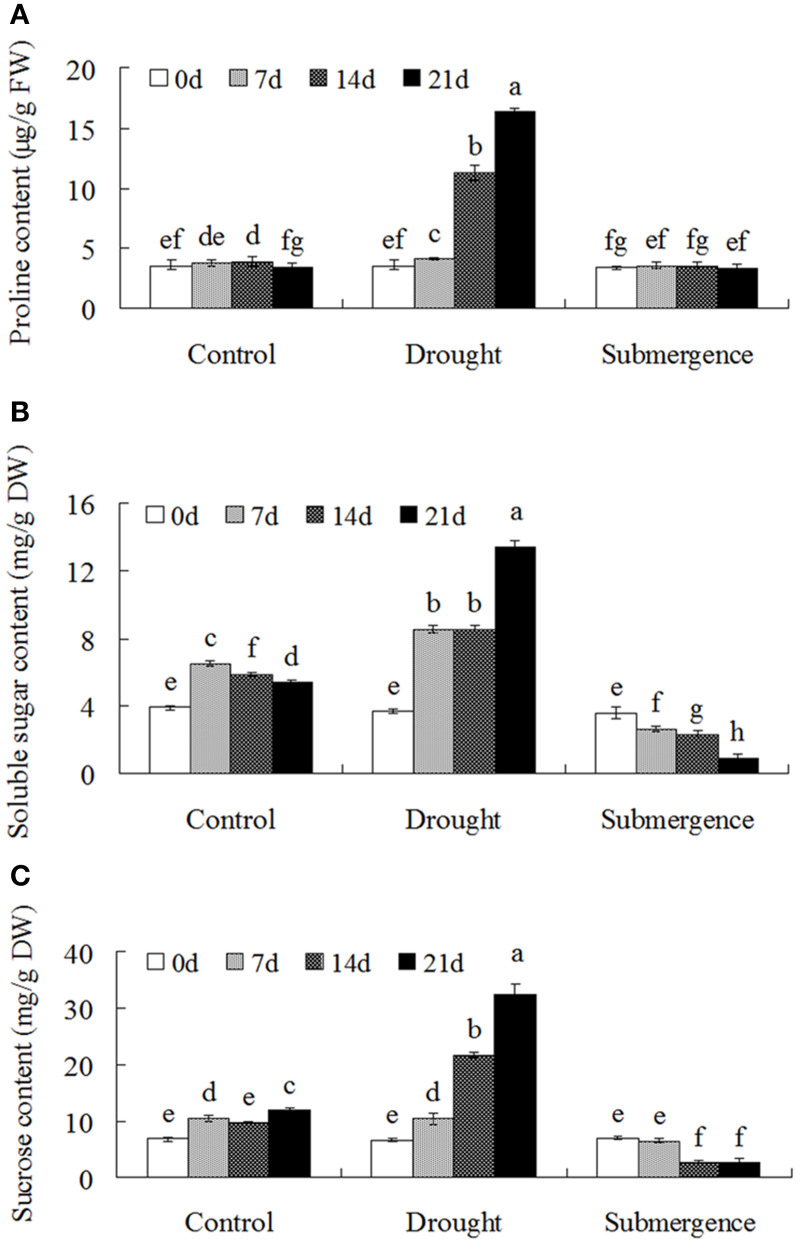
**Osmolytes accumulation of bermudagrass after drought and submergence treatments**. Changes of proline content **(A)**, soluble sugars **(B)**, and sucrose content **(C)** of bermudagrass during control and stressed conditions at indicated days. The results shown are means ± SE (*n* = 4), and the results followed by different letters are significantly different from each other at *P* < 0.05 according to Duncan's method.

Compatible solutes including soluble sugar and proline protect macromolecule structure and at the same time increase the osmotic pressure of the cytoplasm and thereby counteract water loss from cells. Compatible solutes also play key roles during plant redox metabolism (Couee et al., 2006). Under drought condition, proline content increased significantly when compared to the control, but no significant differences were observed in seedlings after submerged (Figure 2A). Interestingly, drought stress treatment significantly increased soluble sugar and sucrose contents in bermudagrass, while submergence caused declined accumulation of soluble sugar and sucrose (Figures 2B,C). These results showed that bermudagrass might develop contrasting strategies to accumulate compatible solute under drought and submergence conditions.

### Protein level changes after drought and submergence treatments

**Figure 3:**
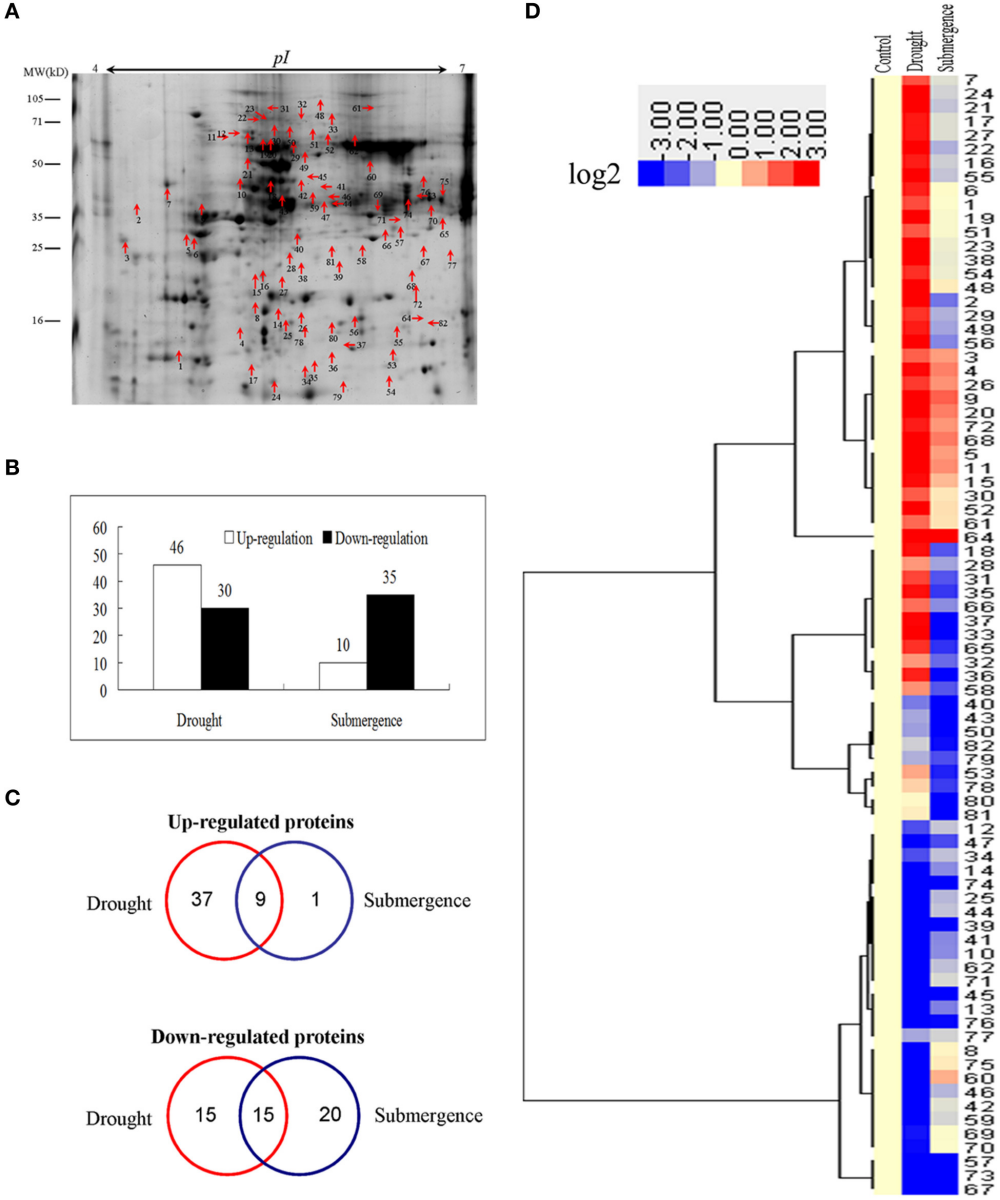
**Proteins changed by drought and submergence**. **(A)** A sketch map to show proteome patterns of bermudagrass in responses to drought and submergence. The protein spots induced at least two folds by drought and submergence were marked with arrows. Proteins were separated in the first dimension on the IPG strip (pH 4–7), and in the second dimension on 12.5% SDS-PAGE. **(B)** Total number of proteins changed by drought and submergence. **(C)** Venn diagram showing the number and of proteins that overlapped among three types of drought and submergence. **(D)** Hierarchical cluster analysis of proteins modulated by drought and submergence treatments. Resulting tree figure was displayed using the software package and Java Treeview. The detailed protein information was listed in Table S1.

To identify proteins simultaneously involved in drought and submergence stress responses in bermudagrass, proteomic analyses based on 2-DE were performed using 14 d stressed samples which showed about 50% EL (Figure 1C). Through proteomics approach, totally 81 proteins regulated by drought or submergence treatment were identified by MALDI-TOF-MS (Figure 3A). Among them, 76 proteins were regulated by drought stress with 46 increased abundance and 30 decreased abundance. Forty-five showed abundance changes after submergence treatment with 10 increased and 35 decreased (Figure 3B). The MS results were matched against NCBInr and Swiss-Port protein sequence databases using MASCOT software, and the best matched protein with high confidence score was selected as the final result of each protein spot (Table S1). Although, Viridiplantae (Green Plants) was chosen as taxonomy during Mascot database search, most putatively identified proteins were matched to those in Poaceae like *Oryza sativa, Triticum urartu, Zea mays*, and *Setaria italica*, which are very close to bermudagrass based on gene sequence alignment analysis.

Overlapping and cluster analyses showed that 9 and 15 proteins were commonly up- and down-regulated by both treatments, respectively (Figures 3C,D). Abundance of 52 and 21 proteins was specifically modulated by drought and submergence stress treatments, respectively (Figures 3C,D). Moreover, we previously identified 27 proteins which showed increased abundances in Yukon after drought treatment. Among them, at least 8 proteins were also significantly up-regulated by drought in this study, including Chitinase, SOD, and heat shock proteins (Table S3).

### Photosynthesis and redox related pathways were enriched after drought and submergence treatment

**Figure 4:**
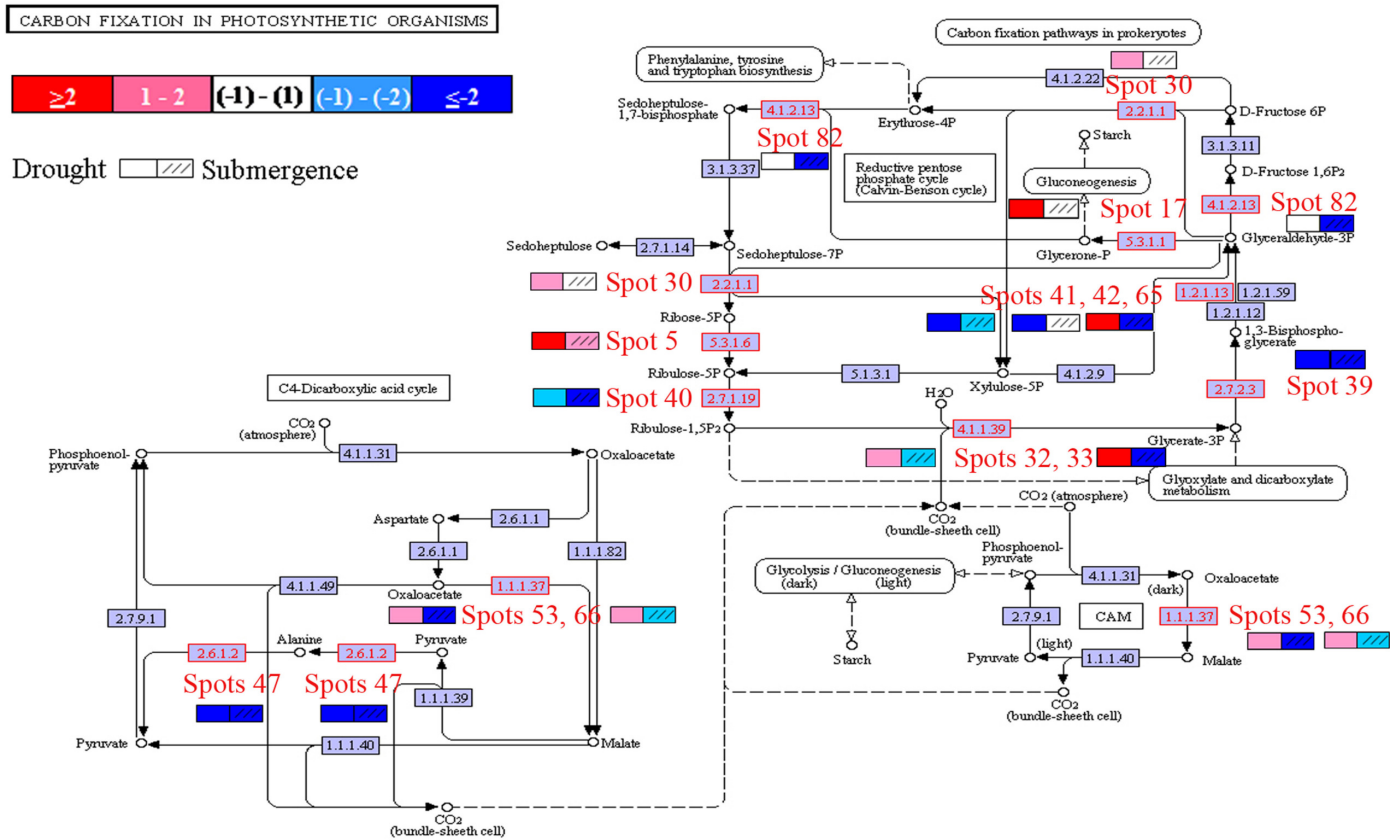
**Characterization of proteins involved in carbon fixation**. Proteins modulated by drought and submergence treatments were involved in carbon fixation in photosynthetic organisms. Specific color code next to each spot represents the log_2_fold changes of drought/control and submergence/control.

Pathway enrichment analysis was then performed. Because of limited reference genome information for bermudagrass, the homologous proteins were blasted against sequenced plant species and functional categories were also assigned using MapMan. The information of homologous protein and functional category of each protein was shown in Table 1 and Table S1. The MapMan pathway enrichment analysis revealed that pathways of amino acid metabolism and mitochondrial electron transport/ATP synthesis were only enriched by drought treatment (Table 1), however, several other pathways including photosynthesis, biodegradation of xenobiotics, oxidative pentose phosphate, glycolysis, and redox were commonly over-represented after both drought and submergence treatments. Further, analysis showed that 14 proteins changed by drought and submergence were involved in carbon fixation in photosynthetic organisms (Figure 4). These results indicated that drought and submergence stresses commonly affected photosynthesis and redox related pathways in bermudagrass.

**Table 1:** **Pathway enrichment analysis of proteins modulated by drought and submergence treatments in bermudagrass**.

**MapMAN pathways**	**Drought**		**Submergence**	
	**NF^a^**	***P-*value**	**NF^a^**	***P*-value**
Photosynthesis	**67.42**	0.0000	**70.92**	0.0000
Biodegradation of xenobiotics	**32.00**	0.0018	**52.17**	0.0007
Oxidative pentose phosphate	**28.90**	0.0021	**47.12**	0.0008
Glycolysis	**28.35**	0.0000	**18.49**	0.0051
Redox	**14.93**	0.0000	**13.91**	0.0002
N-metabolism	**17.23**	0.0450	0.00	0.9650
TCA/org transformation	**17.01**	0.0007	9.24	0.0970
Amino acid metabolism	6.89	0.0025	2.80	0.2510
Mitochondrial electron transport/ATP synthesis	5.93	0.0400	4.83	0.1690
Nucleotide metabolism	2.48	0.2700	0.00	0.7810
Stress	1.09	0.2260	1.18	0.2690
Protein	0.64	0.0650	0.60	0.1020
RNA	0.58	0.0960	0.71	0.1900
Misc	0.56	0.1810	1.37	0.2000
Cell	0.53	0.2910	0.00	0.3160
Transport	0.43	0.2320	0.00	0.2420
Not assigned	0.07	0.0000	0.18	0.0000

a *NF, normalized frequency of each functional category in genome*.

*Black background means NF ≥ 10 and P ≤ 0.05 and gray background means NF ≥ 2 while P ≥ 0.05*.

### Redox and ROS metabolism related proteins were extensively changed after drought and submergence treatments

Since pathways related to redox were largely enriched after drought and submergence treatments, we then checked detailed fold changes of proteins involved in redox and ROS pathways. The results showed that 31 proteins playing key roles during photosynthesis, including RuBisCO activase, Cytochrome b6-f complex, and oxygen-evolving enhancer (Table 2), were mainly induced by drought, but inhibited by submergence treatment. Several redox metabolism related proteins, like peroxidase, and superoxide dismutase showed increased intensities by drought, but decreased intensities by submergence. Dehydrogenase was commonly inhibited by both drought and submergence (Table 2). Chaperonin and heat shock proteins were induced by drought and inhibited by submergence (Table 2). These results showed that redox and ROS related proteins were extensively changed under drought and submergence conditions.

**Table 2:** **Oxidation and redox related proteins changed by drought and submergence stresses in bermudagrass**.

**No**.	**Accession**	**Log**_**2**_ **for fold changes**		**The. Mr(KD)/pI**	**Exp. Mr(KD)/pI**	**Score**	**Sequence coverage %**	**Homologous protein**	**Species**	**Class of bin code +subcategory**
		**D vs. C**.	**Su. vs. C**.							
55	gi|20143564	**3.05**	−0.68	29.4/5.27	47.49/6.06	93	9	ATPase alpha subunit	[*Oryza sativa*]	[1.1] PS.lightreaction
23	gi|150035719	**2.99**	−0.18	35.2/5.52	77.81/5.38	302	62	ATP synthase beta subunit	[*Bouteloua curtipendula*]	[1.1] PS.lightreaction
17	gi|255674406	**2.73**	−0.38	25.8/7.63	26.22/5.26	283	15	PsbP family protein	[*Oryza sativa*]	[1.1] PS.lightreaction
71	gi|225431124	−**3.21**	−0.44	38.8/8.92	34.77/6.89	86	24	Ferredoxin–NADP reductase	[*Vitis vinifera*]	[1.1] PS.lightreaction
73	gi|374411791	−**3.65**	−**3.39**	15.2/5.03	17.4/5.59	60	50	ATP synthase	[*Eleusine coracana*]	[1.1] PS.lightreaction
50	gi|194702912	−**1.12**	−**3.43**	20.7/6.41	9.34/6.27	80	19	Cytochrome b6-f complex	[*Saccharum hybrid*]	[1.1] PS.lightreaction
77	gi|388564565	−**1.01**	−0.52	20.9/8.20	11.53/6.76	128	29	Cytochrome b6-f complex	[*Saccharum hybrid*]	[1.1] PS.lightreaction
74	UCRIA_ORYSJ	−**8.13**	−**2.99**	24.2/8.54	42.23/65.54	84	24	Cytochrome b6-f complex iron-sulfur	[*Oryza sativa*]	[1.1] PS.lightreaction
8	gi|514765640	−**3.29**	0.12	27.4/8.85	17.97/5.11	77	9	Oxygen-evolving enhancer protein 2	[*Setaria italica*]	[1.1] PS.lightreaction
6	gi|161728801	**2.63**	0.04	30.4/5.8	27.07/4.84	149	15	Oxygen evolving protein	[*Oedogonium obesum*]	[1.1] PS.lightreaction
34	gi|131389	−**2.08**	−0.72	25.9/9.14	12.16/5.85	108	16	Oxygen-evolving enhancer	[*Chlamydomonas reinhardtii*]	[1.1] PS.lightreaction
11	gi|357167236	**4.68**	**1.33**	61.2/5.06	66.99/4.98	158	10	RuBisCO binding protein	[*Brachypodium distachyon*]	[1.3] PS.calvin cycle
5	gi|226506270	**4.24**	**1.15**	28.8/5.53	27.78/4.76	114	8	Ribose-5-phosphate isomerase	[*Zea mays*]	[1.3] PS.calvin cycle
19	RUBB_SECCE	**4.08**	−0.07	53.7/4.88	71.0/5.28	138	15	RuBisCO binding protein	[*Secale cereal*]	[1.3] PS.calvin cycle
38	RCA_SOLPN	**3.06**	−0.24	50.9/8.61	45.9/5.82	82	20	RuBisCO activase	[*Solanum pennellii*]	[1.3] PS.calvin cycle
33	gi|321273458	**3.01**	−**3.09**	11.7/5.41	11.93/5.78	118	26	Chloroplast RuBisCO	[*Flaveria trinervia*]	[1.3] PS.calvin cycle
18	gi|13569643	**2.82**	−**2.00**	21.7/4.78	42.33/5.36	138	25	RuBisCO activase	[*Oryza sativa*]	[1.3] PS.calvin cycle
16	gi|113632010	**2.65**	−0.57	32.7/6.96	25.11/5.27	130	11	Triosephosphate isomerase	[*Oryza sativa*]	[1.3] PS.calvin cycle
30	gi|113594751	**1.91**	0.28	74.0/5.44	72.55/5.51	80	13	Transferas	[*Oryza sativa*]	[1.3] PS.calvin cycle
58	gi|424949905	**1.28**	−**1.98**	51.7/6.14	63.57/6.16	75	24	RuBisCO large subunit	[*Bromus inermis*]	[1.3] PS.calvin cycle
32	gi|13241107	**1.20**	−**1.74**	15.6/8.24	11.45/5.67	133	16	RuBisCO	[*Flaveria palmeri*]	[1.3] PS.calvin cycle
82	gi|514807945	−0.56	−**2.87**	35.9/5.35	34.60/6.48	107	31	Fructose-bisphosphate aldolase	[*Setaria italica*]	[1.3] PS.calvin cycle
43	RCA_PHAAU	−**1.02**	−**3.70**	48.0/7.57	42.7/5.80	134	11	RuBisCO activase	[*Phaseolus aureus*]	[1.3] PS.calvin cycle
40	gi|115448091	−**1.54**	−**5.69**	45.2/5.68	72.75/5.67	132	20	Phosphoribulokinase (PRK)	[*Oryza sativa*]	[1.3] PS.calvin cycle
12	gi|134102	−**2.07**	−0.68	57.7/4.83	68.26/5.16	135	9	RuBisCO binding protein	[*Triticum aestivum*]	[1.3] PS.calvin cycle
13	gi|406034834	−**3.11**	−**1.49**	52.5/6.14	64.01/5.15	236	26	RuBisCO	[*Neyraudia reynaudiana*]	[1.3] PS.calvin cycle
62	gi|340511244	−**5.08**	−0.73	49.3/6.34	28.65/6.62	186	26	RuBisCO large subunit	[*Eleusine indica*]	[1.3] PS.calvin cycle
75	gi|290586402	−**6.16**	0.23	26.4/7.78	15.13/5.81	170	32	RuBisCO large subunit	[*Acorus calamus*]	[1.3] PS.calvin cycle
45	gi|452029828	−**8.87**	−**5.40**	48.2/6.33	58.6/5.63	176	27	RuBisCO large subunit	[*Digitaria radicosa*]	[1.3] PS.calvin cycle
10	gi|13569643	−**9.12**	−**1.35**	21.7/4.78	47.95/5.17	213	22	RuBisCO activase	[*Oryza sativa*]	[1.3] PS.calvin cycle
59	gi|339759470	−**10.31**	−0.47	5.2/5.06	7.77/6.73	107	72	RuBisCO small subunit protein	[*Eleusine coracana*]	[1.3] PS.calvin cycle
65	gi|514801897	**2.42**	−**2.41**	42.9/6.60	34.43/6.84	360	13	Dehydrogenase A	[*Setaria italica*]	[1.3] PS.calvin cycle
42	gi|113547318	−**6.39**	−0.35	47.5/6.22	44.85/5.85	188	12	GAP dehydrogenase	[*Oryza sativa*]	[1.3] PS.calvin cycle
41	G3PA_CHLRE	−**5.06**	−**1.39**	40.5/9.17	48.9/5.68	78	18	Dehydrogenase A	[*Chlamydomonas reinhardtii*]	[1.3] PS.calvin cycle
49	gi|413923213	**2.67**	−**1.05**	17.1/9.97	10/6.37	106	10	Ferredoxin-thioredoxin reductase	[*Zea mays*]	[21.1] Redox.thioredoxin
64	gi|218195985	**3.40**	**4.11**	17.4/5.56	27.21/6.59	98	22	Dehydroascorbate reductase	[*Oryza sativa*]	[21.2] Redox.ascorbate and glutathione
80	gi|338760827	0.07	−**3.99**	27.5/5.79	25.34/5.92	371	33	Ascorbate peroxidase	[*Eleusine coracana*]	[21.2] Redox.ascorbate and glutathione
36	gi|514737330	**2.62**	−**4.05**	27.2/5.18	25.26/5.6	77	16	L-ascorbate peroxidase 2	[*Setaria italica*]	[21.2] Redox.ascorbate and glutathione
1	gi|473825951	**2.10**	−0.02	26.3/6.32	11.65/4.69	81	11	Peroxiredoxin-2E-1	[*Triticum urartu*]	[21.5] Redox.peroxiredoxin
51	gi|301073308	**1.97**	−0.07	15.3/5.65	11.81/6.32	96	20	Cu/Zn superoxide dismutase	[*Aeluropus lagopoides*]	[21.6] Redox.dismutases and catalases
69	gi|475575990	−**2.77**	−0.05	33.9/9.22	46.68/6.75	69	15	Peroxidase 12	[*Aegilops tauschii*]	[26.12] Misc.peroxidases
76	gi|475582712	−**6.03**	−**2.92**	31.2/8.84	28.23/5.92	209	15	Peroxidase 70	[*Aegilops tauschii*]	[26.12] Misc.peroxidases
44	gi|347602486	−**3.19**	−0.71	101.8/6.14	90.3/5,81	278	30	Chaperone protein	[*Oryza sativa*]	[29.5] Protein.degradation
20	gi|514759641	**3.39**	**1.52**	62.1/5.43	60.95/5.37	187	13	Chaperonin 60 subunit beta 2	[*Setaria italica*]	[29.6] Protein.folding
4	gi|256708477	**2.92**	**1.48**	27.7/6.71	17.43/4.88	167	15	Chitinase	[*Leymus chinensis*]	[20.1] Stress.biotic
22	HSP7C_PETHY	**3.56**	−0.91	71.6/5.11	75.22/5.29	156	29%	Heat shock cognate 70	[*Petunia hybrid*]	[20.2] Stress.abiotic
52	HSP7M_PEA	**3.25**	0.36	72.4/5.81	17.06/6.17	69	18%	Heat shock 70 kDa protein	[*Pisum sativum*]	[20.2] Stress.abiotic.heat

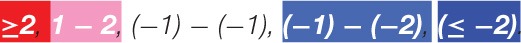

### Modulation of ROS metabolism in bermudagrass after drought and submergence treatments

**Figure 5:**
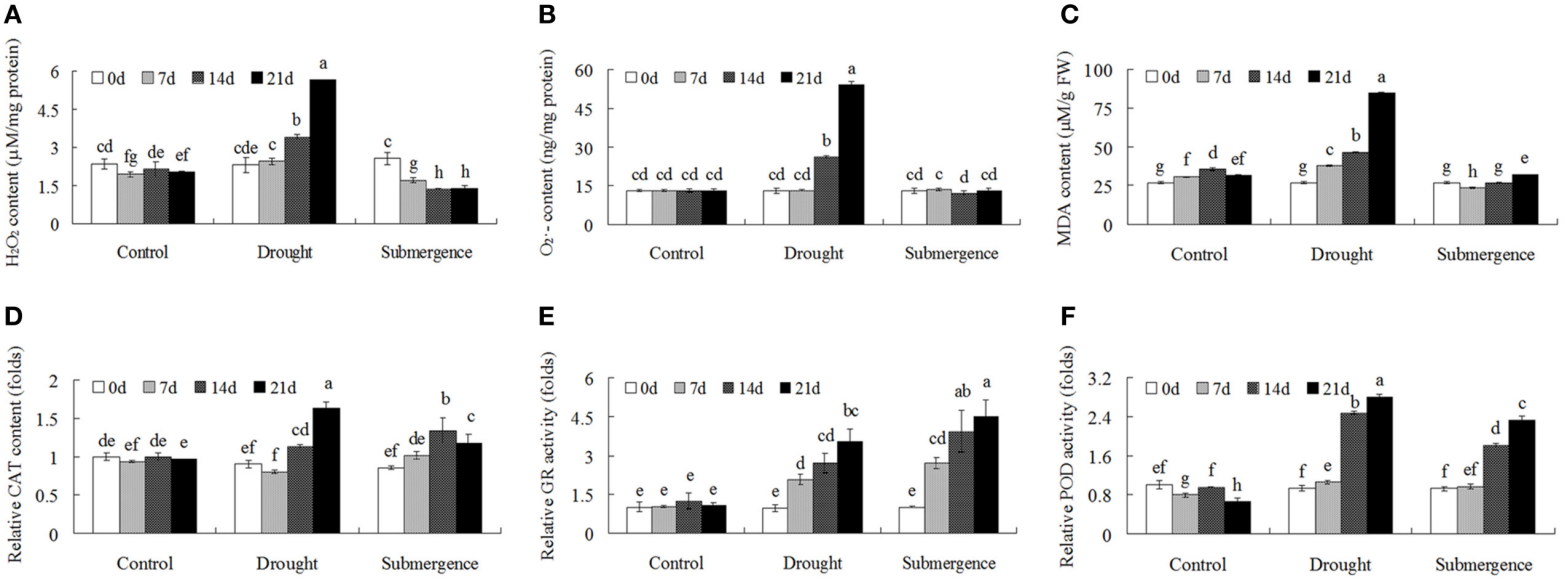
**Changes of ROS level and antioxidant enzyme activities after drought and submergence treatments**. Changes of H_2_O_2_ content **(A)**, ${\text{O}}_{2}^{∙-}$ content **(B)**, and MDA level **(C)** of bermudagrass at indicated timepoints. Changes of CAT **(D)**, GR **(E)**, and POD **(F)** activities under stress conditions at designated time intervals. The relative activities were quantified as fold change in comparison with bermudagrass under control condition. The data represent the means of three independent experiment ± SE, and data followed by different letters are significantly different from each other at *P* < 0.05 according to Duncan's method.

To further investigate ROS homeostasis caused by drought and submergence stresses, the detailed content changes of reactive oxygen species were determined. After drought treatment, both H_2_O_2_ and ${\text{O}}_{2}^{-}$ contents increased after 14 d stress treatment. However, under submergence condition, H_2_O_2_ content decreased and ${\text{O}}_{2}^{-}$ content showed no significant changes (Figures 5A,B). MDA is one of the most frequently used indicators of lipid peroxidation, and MDA content reflects the degree of membrane lipid peroxidation. Drought treatment significantly increased MDA content while submergence slightly increased MDA content (Figure 5C). Antioxidant enzymes activities, including CAT, GR, and POD, were then analyzed to reveal changes of enzymatic defense systems. Both drought and submergence treatments increased CAT, GR, and POD activities (Figures 5D–F). These results indicated that drought and submergence treatments modulated antioxidant enzyme activities and caused contrasting ROS content changes in bermudagrass.

### Modulation of metabolites in bermudagrass after drought and submergence treatments

**Figure 6:**
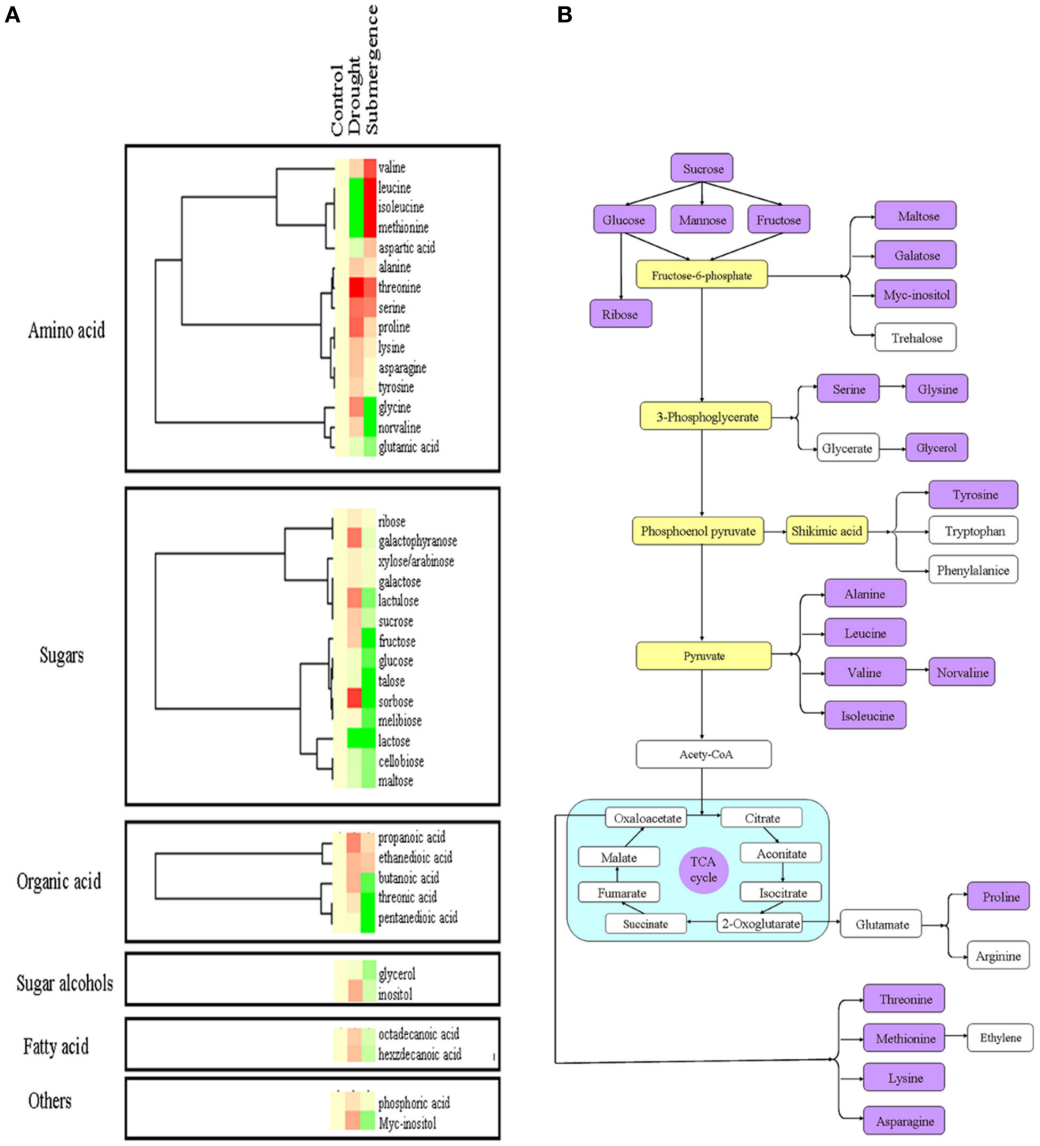
**Effects of drought and salt stresses on metabolites in bermudagrass**. **(A)** Hierarchical cluster analysis of 40 compounds affected by drought and submergence stresses. The resulting tree figure was obtaining using the Java Treeview and the CLUSTER software package. **(B)** Assignment of the 19 metabolites studied to pathways. A total of 19 metabolites were indicated in boxes with rose red colors, and the concentrations of these metabolites were shown in Table S2.

Since several proteins involved in carbon fixation were changed after stress treatments (Figure 4), primary metabolite contents were then determined through chromatography time-of-flight mass spectrometry (GC-TOF-MS). In total, 40 metabolites were measured, including 15 amino acids, 14 sugars, 5 organic acid, 2 sugar alcohols, 2 fatty acid and 2 others (Figures 6A, 4B; Table S2). After drought and submergence treatments, contents of most amino acid increased, including theronine, serine, and proline. However, contents of most sugars, organic acid, sugar alcohols, and fatty acid increased by drought, but decreased by submergence. Among 40 metabolites, 22 metabolites involved in carbon and amino acid metabolic pathways (Figure 6B) were commonly modulated by drought and submergence stresses, further confirming the carbon and amino acid metabolisms were extensively changed in response to abiotic stresses.

## Discussion

Plants periodically exposed to drought and submergence stresses in field condition which greatly inhibited plant growth, development and production. Abiotic stresses trigger complex signaling transduction pathways which may lead to an imbalance between antioxidant defenses and the amount of ROS, resulting in oxidative stress (Pastori and Foyer, 2002; Xiong et al., 2002). ROS are harmful by-products of normal cellular metabolism in aerobic organisms (Apel and Hirt, 2004; Miller et al., 2010) and can directly attack membrane lipids, resulting in lipid peroxidation and oxidation of proteins and nucleic acids (Kranner et al., 2010; Alhdad et al., 2013). In addition to the toxicity of ROS, ROS are necessary for inter- and intracellular signaling and considered to be signaling molecules that regulate plant growth and development, adaptation to abiotic and biotic stress factors (Apel and Hirt, 2004; Mittler et al., 2004). To scavenge ROS, plants have evolved an efficient enzymatic and non-enzymatic antioxidative system to protect themselves against oxidative damage and fine modulation of low levels of ROS for signal transduction. Enzymatic antioxidants in plant include SOD, CAT, POD, GR, DHAR, GST, and PRX (Miller et al., 2010; Meyer et al., 2012; Noctor et al., 2014). Non-enzymatic antioxidants including glutathione (GSH), ascorbic acid (AsA), carotenoids, tocopherols, and flavonoids are also crucial for ROS homeostasis in plant (Gill and Tuteja, 2010). In this study, enzyme activities of POD, CAT, and GR increased after drought and submergence treatments (Figure 5), while protein abundances of SOD, POD, and PRX were enhanced by drought but inhibited by submergence (Table 2). However, H_2_O_2_, ${\text{O}}_{2}{∙}^{-}$ and MDA contents increased only after drought treatment (Figure 5), and no significant changes were found for submerged bermudagrass (Figure 5). These results showed that bermudagrass under drought condition suffered from oxidative stress while submerged plants did not.

Besides traditional enzymatic and non-enzymatic antioxidants, increasing evidences indicated that soluble sugars have a dual role with respect to ROS (Couee et al., 2006; Keunen et al., 2013). Soluble sugars were directly linked with the production rates of ROS by regulation ROS producing metabolic pathways, such as mitochondrial respiration or photosynthesis. Conversely, they also feed NADPH-producing metabolism such as the oxidative pentose-phosphate pathway to involved in antioxidative processes (Couee et al., 2006). Drought stress caused significant increases of soluble sugars and sucrose (Figure 2). Proteomic analysis also revealed that 14 proteins involved in photosynthesis and carbon fixation were highly induced under drought condition (Figure 4; Table 2). These data was confirmed by metabolomic results which showed that sugars, organic acid, sugar alcohols, and fatty acid increased after drought treatment (Figure 6). However, only slight changes were observed after submergence treatment. Proline, acting as osmoprotectors, protects protein structures from stress caused damages. Proline also functions as a ROS scavenger, especially for hydroxyl radical (Smirnoff and Cumbes, 1989). Higher proline content in plants has been shown to be associated with increased tolerance to oxidative stress (Arbona et al., 2008). In this study, drought stress increased proline content in bermudagrass while submergence had no significant effect on proline accumulation (Figures 2, 6). Taken together, the decreases or insignificant changes of 85% metabolites in submerged bermudagrass may be probably related to its physiological dormancy encountered deep submergence stress (Gibbs and Greenway, 2003; Bailey-Serres and Voesenek, 2008).

Photosynthesis has a high capacity for production of ROS. The primary event of photosynthesis is light-driven electron transfer–a redox reaction. During photosynthesis, electrons produced from water are transferred from the reaction center of photosystem II (PSII) to the cytochrome *b*6*f* (Cyt *b*6*f*) complex by the mobile electron carrier plastoquinone (PQ). Electrons from the cytochrome *b6f* complex are then transferred to photosystem I (PSI) by plastocyanin (PC). Under adverse environmental condition, electrons of PSI can also be transferred to oxygen, which results in the generation of ROS (Pfannschmidt et al., 2001; Pfannschmidt, 2003). Three proteins identified as Cyt *b*6*f* were inhibited by both drought and submergence in bermudagrass (Table 2), indicating that transfer of electrons from PS II to PS I became impaired. In addition, ATP synthase and ATPase showed more than 8-fold increases only by drought treatment in bermudagrass (Table 2). These results indicated that both drought and submergence affected photosynthesis, however, drought promoted while submergence declined ATP biosynthesis. Moreover, 7 RuBisCO related proteins showed 2.3–25.6 folds intensity change in bermudagrass after drought treatment (Table 2). RuBisCO is involved in the first key step of carbon fixation during calvin cycle. These data verified that photosynthesis was promoted by drought, but inhibited after submergence.

Several amino acids such as leucine, isoleucine, methionine were significantly increased after submergence treatment, but decreased after drought stress (Figure 6). For example, the content of methionine using for ethylene synthesis was significantly induced by submergence stress, but decreased by drought stress. The ethylene accumulation is very important for plants to cope with submergence stress (Hattori et al., 2009; Niroula et al., 2012). In addition, some carbohydrates such as glucose, sucrose, sorbose, melibiose, and fructose were significantly down-modulated by submergence. Therefore, the bermudagrass could develop specific mechanism such as restriction of carbohydrate consumption and ethylene accumulation to cope with submergence stress during physiological dormancy period. Therefore, under submergence condition, bermudagrass may invoke a quiescence strategy with repressed carbohydrate metabolism and retarded cell elongation. This hypothesis was confirmed by completely inhibited growth after submerged (Figure 1).

It has been reported that waterlogging reduced biomass in cool-season creeping bentgrass (Huang et al., 1998; Jiang and Wang, 2006) and Kentucky bluegrass (Wang and Jiang, 2007), as well as in warm-season seashore paspalum and centipedegrass (Zong et al., 2015). However, waterlogging stimulated plant growth in other warm-season grass species such as knotgrass and spiny mudgrass (Zong et al., 2015). According to the field survey results in the Three Gorges Reservoir in China, bermudagrass can tolerate deep and prolonged submergence stress for half a year (Tan et al., 2010). Through, physiological analysis, we observed many parameters showed significant changes after drought treatment, while only slight changes after submergence treatment, including osmolytes accumulation and ROS level and antioxidant enzyme activities (Figures 2, 5). Proteomics results showed that abundance of only 10 proteins increased by submergence, while 46 proteins by drought (Figure 3). Metabolomic analysis indicated that most of the metabolites were up-regulated by drought stress, while 34 of 40 metabolites contents exhibited down-regulation or no significant changes when exposed to submergence stress (Figure 6). These data were consistent with results observed by Tan et al. (2010) that submergence decreased total soluble carbohydrate and starch contents in bermudagrass. As reported previously (Shi et al., 2014), 27 proteins were induced by drought in Yukon leaf and 8 of them were identified to be drought stress inducible in this study, including, chitinase, SOD, and heat shock proteins (Table S3). All these data indicated that ROS and stress related proteins played important role during bermudagrass stress response.

**Figure 7:**
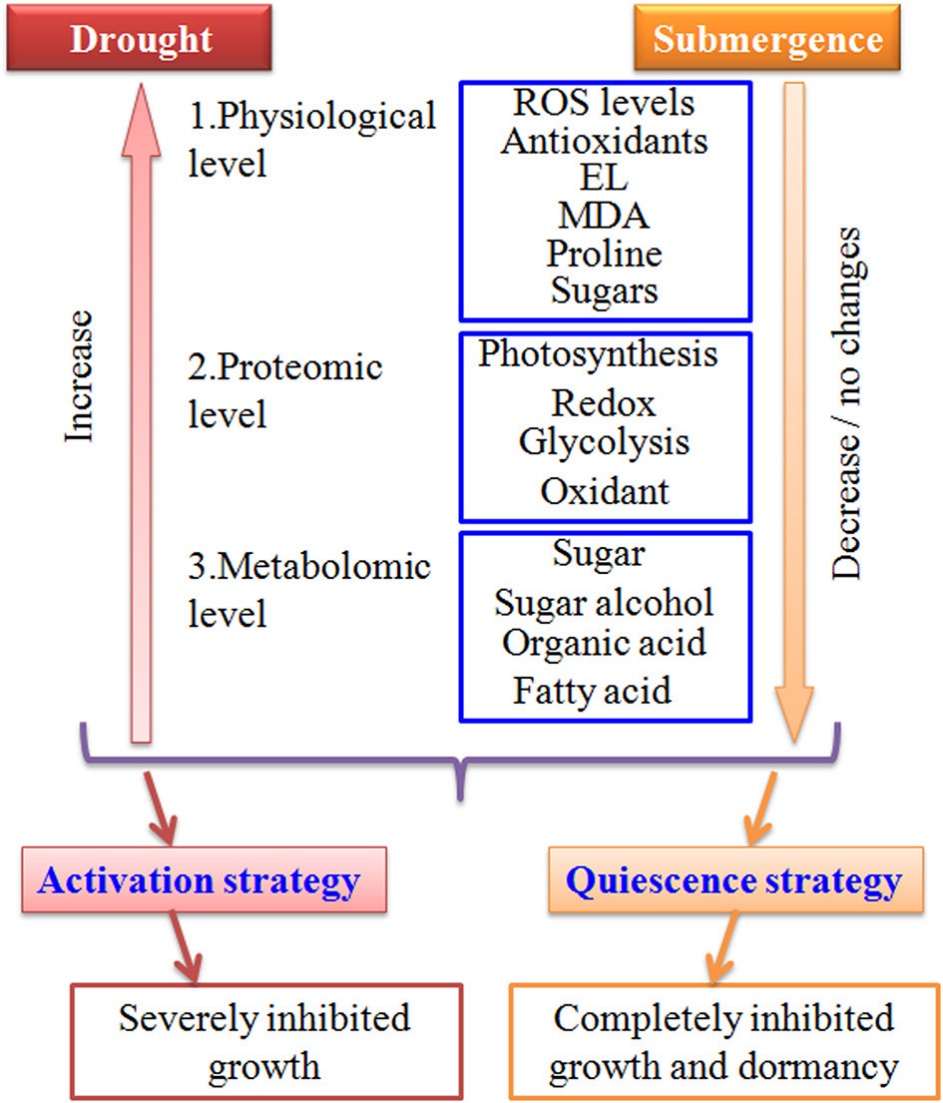
**Model depicts drought and submergence caused contrasting changes in bermudagrass**. Bermudagrass developed drought stress tolerance through activation of physiological, proteomic, and metabolomic pathways, resulting in accumulation of compatible solutes and molecular chaperones. However, bermudagrass may invoke a quiescence strategy with repressed carbohydrate metabolism and retarded cell elongation. Therefore, drought severely inhibited while submergence completely inhibited bermudagrass growth.

In conclusion, bermudagrass might slow down metabolisms such as carbonhydrate degradation and energy supply under submergence stress, resulting in completely inhibited growth (Figures 1, 7). The quiescence strategy with retarded growth might allow bermudagrass to be adaptive to long-term submerged environment. However, bermudagrass developed drought stress tolerance through activation of photosynthesis and redox, leading to accumulation of compatible solutes and molecular chaperones (Figure 7).

### Conflict of interest statement

The authors declare that the research was conducted in the absence of any commercial or financial relationships that could be construed as a potential conflict of interest.

## Supplementary material

Figure S1 **The detailed design of the experiment**.

Table S1 **Details of different expression**.

Table S2 **Quantitive value mg per g F.W**.

Table S3 **Comparison of the proteomic results with the previous research (Shi et al., 2014)**.

## Abbreviations

2-DE: two-dimensional gel electrophoresis
ABA: abscisic acid
CAT: catalase
DHAR: dehydroascorbate reductase
DW: dry weight
EL: Electrolyte Leakage
FW: fresh weight
GA: gibberellic acid
GR: glutathione reductase
GST: glutathione S-transferase
IEF: isoelectric focus
LWC: leaf water content
MDA: malondialdehyde
PC: plastocyanin
POD: Peroxidase
PQ: plastoquinone
PRX: peroxiredoxin
PSI: photosystem I
PSII: photosystem II
ROS: reactive oxygen species
RuBisCO: Ribulose-1,5-bisphosphate carboxylase/oxygenase
SDS-PAGE: SDS polyacrylamide gel electrophoresis.
