# Supplementary figures and images for: Contrasting Changes Caused by Drought and Submergence Stresses in Bermudagrass (Cynodon dactylon)
Source: Front Plant Sci. 2015 Nov 10;6:951. doi: 10.3389/fpls.2015.00951 (PMC4639625; doi:10.3389/fpls.2015.00951)

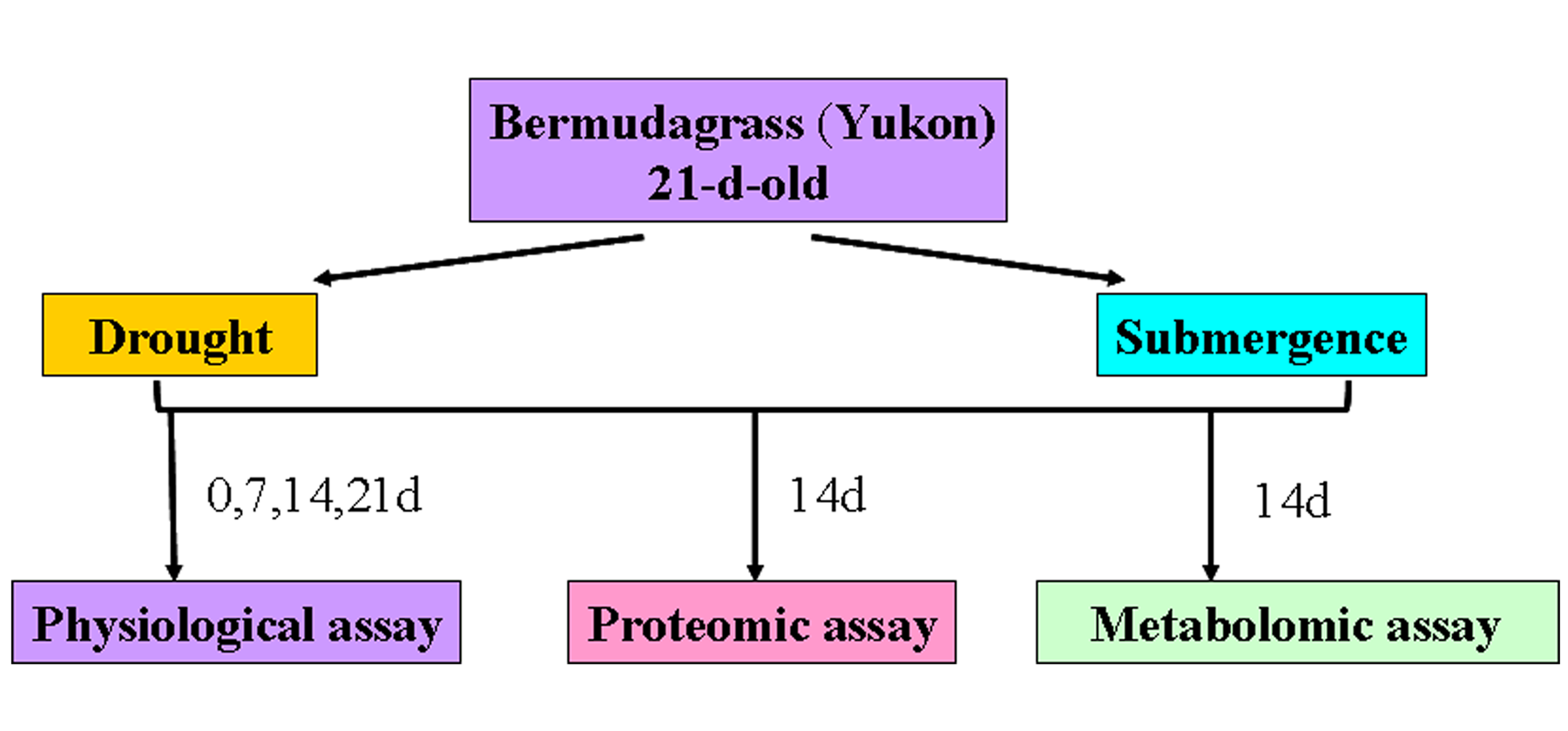

Supplement: Figure S1 — The detailed design of the experiment. [file Image1.TIF]
